# Green tea catechin-grafted silk fibroin hydrogels with reactive oxygen species scavenging activity for wound healing applications

**DOI:** 10.1186/s40824-022-00304-3

**Published:** 2022-11-09

**Authors:** Gyeongwoo Lee, Young-Gwang Ko, Ki Hyun Bae, Motoichi Kurisawa, Oh Kyoung Kwon, Oh Hyeong Kwon

**Affiliations:** 1 Department of Polymer Science and Engineering, Kumoh National Institute of Technology, Gumi, Gyeongbuk 39177 Korea; 2 Institute of Bioengineering and Bioimaging, 31 Biopolis Way, The Nanos, Singapore 138669, Singapore; 3 Gastrointestinal surgery, Kyungpook National University Chilgok Hospital, Daegu 41404, Korea; 4 Department of Surgery, Kyungpook National University School of Medicine, Daegu 41944, Korea

**Keywords:** Silk fibroin, EGCG, Hydrogel, Reactive oxygen species, Wound healing

## Abstract

**Background:**

Overproduction of reactive oxygen species (ROS) is known to delay wound healing by causing oxidative tissue damage and inflammation. The green tea catechin, (–)-Epigallocatechin-3-*O*-gallate (EGCG), has drawn a great deal of interest due to its strong ROS scavenging and anti-inflammatory activities. In this study, we developed EGCG-grafted silk fibroin hydrogels as a potential wound dressing material.

**Methods:**

The introduction of EGCG to water-soluble silk fibroin (SF-WS) was accomplished by the nucleophilic addition reaction between lysine residues in silk proteins and EGCG quinone at mild basic pH. The resulting SF-EGCG conjugate was co-crosslinked with tyramine-substituted SF (SF-T) *via* horseradish peroxidase (HRP)/H_2_O_2_ mediated enzymatic reaction to form SF-T/SF-EGCG hydrogels with series of composition ratios.

**Results:**

Interestingly, SF-T70/SF-EGCG30 hydrogels exhibited rapid in situ gelation (< 30 s), similar storage modulus to human skin (≈ 1000 Pa) and superior wound healing performance over SF-T hydrogels and a commercial DuoDERM® gel dressings in a rat model of full thickness skin defect.

**Conclusion:**

This study will provide useful insights into a rational design of ROS scavenging biomaterials for wound healing applications.

**Supplementary Information:**

The online version contains supplementary material available at 10.1186/s40824-022-00304-3.

Running head: Green tea catechin-grafted silk fibroin hydrogel wound dressing.

## Introduction

Reactive oxygen species (ROS) are messenger molecules playing pivotal roles in multiple wound healing processes, including cell migration, angiogenesis and epithelialization [1]. Emerging evidence suggests that elevated levels of ROS cause impaired wound healing through creating redox imbalance, oxidative stress and persistent inflammation [2]. For example, neutrophils and macrophages recruited at a wound lesion have been shown to release ROS, such as superoxide anion radical (O_2_•¯) and hydroxyl radical (•OH), which can drive apoptotic death of dermal fibroblasts and inhibit the proliferation of keratinocytes and their migration towards the wound area, thus disturbing re-epithelialization for proper wound closure [3, 4]. Moreover, an aberrant overproduction of ROS has been reported to stimulate dermal fibroblasts to secrete matrix metalloproteinases, which facilitate uncontrolled destruction of the extracellular matrix and thus delay wound repair processes [5, 6]. This sequence of events accelerates the recruitment of leukocytes and secretion of proinflammatory cytokines, further exacerbating the severity of wound trauma [7]. In this perspective, ROS scavenging biomaterials capable of attenuating oxidative stress can be potentially beneficial for promoting wound healing process.

Silk fibroin (SF) is a natural protein extracted from *Bombyx mori* silk worms, which is composed of over 5,000 amino acids in length [8]. Owing to its desirable characteristics including high flexibility, biocompatibility and biodegradability, SF has been widely used to design biomaterials for reconstruction of diverse soft tissues, such as skin, cornea, nerve, ligament, and articular cartilage [9, 10]. To date, significant efforts have been devoted to physical and chemical modifications of SF-based materials for the enhancement of their physicochemical, mechanical and biological properties. For instance, SF scaffold has been modified with collagen [11], chondroitin sulfate [12] and chitosan [13] to support chondrogenesis and cartilage repair. Crosslinking SF fibers with elastin-like polypeptides containing repeated blocks of (Val-Pro-Gly-Xaa-Gly)_n_ has been shown to promote the attachment and osteogenic differentiation of bone marrow stem cells [14]. The introduction of graphene oxide into SF/nanohydroxyapatite scaffolds enhanced their elastic modulus, physiological stability and bone regeneration rates [15]. Tannic acid, a plant-derived natural polyphenol, has been incorporated into SF hydrogels to endow them with cell adhesiveness, antimicrobial and antioxidant properties [16]. Especially for wound healing, SF has received immense attention because of its ability to promote fibroblast proliferation, angiogenesis and extracellular matrix deposition with beneficial for wound repair and skin reconstruction process [17 – 24]. Recently, highly elastic and resilient SF hydrogels have been developed by horseradish peroxidase (HRP)/H_2_O_2_-mediated enzymatic crosslinking of tyrosine residues in silk proteins [25, 26]. In addition, tyramine-substituted SF (SF-T) has been blended with native SF to fabricate hybrid SF-T/SF hydrogels with tunable mechanical strength, gelation kinetics and swelling properties [27].

In spite of the potential benefits for wound healing applications, development of ROS scavenging SF hydrogels has remained largely unexplored. (–)-Epigallocatechin-3-*O*-gallate (EGCG), the major catechin extracted from green tea, has long been recognized as a powerful ROS scavenger and anti-inflammatory agent [28, 29]. EGCG has also been shown to induce the differentiation of both young and aged keratinocytes, highlighting its potential to expedite skin regeneration [30]. From this perspective, introduction of EGCG into SF based hydrogels can be advantageous for improving their wound healing performance. In this study, we report the development of EGCG-grafted SF composite hydrogels with ROS scavenging activity as a potential wound dressing material. SF-EGCG conjugate was synthesized by the nucleophilic addition reaction between lysine residues in silk proteins and EGCG quinone under mild alkaline condition.

This research demonstrated the chemical modification, cytocomaptibility, and ability of SF-EGCG conjugates to scavenge two different types of ROS: O_2_•¯ and •OH which are known to be involved in the elevated oxidative stress in wound sites [7]. SF-T/SF-EGCG composite hydrogels with different composition ratios were prepared by HRP/H_2_O_2_-mediated enzymatic crosslinking reaction and characterized for their storage modulus, gelation time, swelling behavior and stability in physiological environment. The potential feasibility of the composite hydrogels as wound dressing materials was explored in a rat model of full-thickness skin defect.

## Experimental section

### Materials and reagents

Refined *Bombyx mori* silk fibroin was obtained from Dasung Silk (Korea). Lithium bromide monohydrate (LiBr) and xanthine were purchased from Daejung Metals and Chemical (Korea). (–)-Epigallocatechin-3-*O*-gallate (EGCG, pharmaceutical secondary standard), tyramine hydrochloride, *N*-hydroxysuccinimide (NHS), 2-(*N*-morpholino)ethanesulfonic acid (MES), L-cysteine, 5,5`-dithiobis(2-nitrobenzoic acid) (DTNB), fluorescamine, 2-deoxy-D-ribose, trichloroacetic acid, thiobarbituric acid, xanthine oxidase from cow milk, horseradish peroxidase (HRP, Type II, specific activity: 251 unit/mg) were purchased from Sigma-Aldrich (USA). Ascorbic acid and iron (II) sulfate (FeSO_4_) were purchased from Junsei (Japan). Glycine, 1-(3-dimethylaminopropyl)-3-ethylcarbodiimide hydrochloride (EDC) and 4-nitro blue tetrazolium chloride (NBT) were purchased from TCI (Japan). Enzchek™ gelatinase/collagenase assay kit was purchased from Thermofisher (USA). All reagents were of the highest purity and used without further purification.

### Preparation of water-soluble silk fibroin (SF-WS) with molecular weight analysis

Silk fibroin was regenerated into a water-soluble form by thermal hydrolysis method [31]. As first procedure, refined *Bombyx mori* silk fibroin (30 g) was dissolved in 9.3 M LiBr aqueous solution (500 mL) and vigorously stirred for 7 h (100ºC). After that, the homogenous solution was dialyzed against purified water for 72 h (MWCO: 14,000), with dialysate exchange every 12 h. The dialyzed solution was filtered by a filter paper (pore size: 4 μm) to eliminate impurities. The resulting water-soluble silk fibroin (SF-WS) solution was lyophilized to obtain a dry product.

To analyze the molecular weight, SF-WS was dissolved in 0.1 M NaNO_3_ at a concentration of 10 mg/mL and filtered with a syringe filter (pore size: 0.45 μm). Subsequently, 200 µL of SF-WS solution was injected into the multiple column system of EcoSEC HLC-8320 GPC (Tosoh, Japan) equipped with TsKgel guard PWxl, Tskgel GMPWxl, TSKgel G2500PWxl column (7.8 mm × 300 mm) and a refractive index detector. The mobile phase (0.1 M NaNO_3_) was delivered at a flow rate of 1 mL/min at 40°C. The number-average molecular weight (*M*_n_) and weight-average molecular weight (*M*_w_) were calculated using polyethylene oxide (PEO) narrow-molecular-weight calibration standards.

### Conjugation of tyramine onto SF-WS

SF-WS was modified with tyramine through a carbodiimide coupling reaction using EDC and NHS according to the previous report [27]. Briefly, regenerated SF-WS (3 g) was dissolved in 40 mL of purified water, and mixed with 160 mL of 62.5 mM MES buffer solution (pH 6.0). Subsequently, 6 g of tyramine hydrochloride, 6 g of EDC and 1.5 g of NHS were added to SF-WS solution and stirred for 12 h (25ºC, 150 RPM). The resulting tyramine-substituted SF (SF-T) solution was dialyzed against 100 mM NaCl solution for 48 h and against purified water for additional 48 h. The dialyzed solution was filtered by a syringe filter (pore size: 0.45 μm) and then lyophilized to obtain a dry product.

### Conjugation of EGCG onto SF-WS

SF-WS was modified with EGCG according to the previously reported method with some modifications [32]. Briefly, SF-WS (1 g) was dissolved in 140 mL of PBS solution (pH 7.4), and mixed with 60 mL of PBS solution containing various feeding amounts of EGCG (10, 20, 40, 60, 80, 120 µmol). The pH of the mixture solution was adjusted to 7.4 and stirred for various periods of time up to 5 h (25ºC, 250 RPM). Afterwards, the pH of the mixture solution was adjusted to 6.0 to terminate the reaction and then dialyzed against purified water for 72 h under nitrogen atmosphere (dialysate exchanged every 12 h). The dialyzed mixture solution was filtered by a syringe filter (pore size: 0.45 μm). The EGCG-modified silk fibroin (SF-EGCG) solution was lyophilized to obtain a dry product.

### Chemical characterization of SF-WS, SF-T and SF-EGCG

Chemical structures of SF-WS and SF-T were investigated by proton nuclear magnetic resonance spectroscopy (^1^ H NMR, Bruker Biospin Advance III, Germany). SF-WS and SF-T were dissolved in deuterium oxide (D_2_O) at a concentration of 5 mg/mL for NMR measurement. The integrated intensity of the characteristic peaks of tyrosine and tyramine moieties (6.6 ~ 7.5 ppm, B1, B2) was compared with that of the peak of alanine residues comprising 30.3% of silk fibroin (1.3 ~ 1.5 ppm, A) for each sample to determine the degree of substitution (DS, defined as the number of substituents per 100 amino acid residues of SF-WS).

The extent of EGCG modification was determined by comparing the difference in absorbance at 274 nm between SF-EGCG and SF-WS with a series of EGCG standard solutions. SF-WS and SF-EGCG were dissolved in purified water for UV-visible spectroscopy (OPTIZEN 3220UV, Mecasys, Korea).

The chemistry between EGCG and the free amine group was observed by mass spectrometry. Briefly, EGCG and L-lysine or ethanolamine were dissolved in the distilled water at a concentration of 0.4 mM. After that, pH was adjusted to 7.4 and stirred for 4 h. After the stirring, pH was adjusted to 6.0, and the solution was purged with nitrogen. After 24 h, the solution was measured using Xevo TQ-S micro mass spectrometer (Waters, USA). 0.1% formic acid and 0.1% acetonitrile mixture were used as mobile phase, and the flow rate of the sample was 0.2 ml/min. Measurement was conducted under 1.5 kV of capillary voltage, 20 V of cone voltage, and 150ºC of source temperature, and positive electrospray ionization mode was observed.

The cysteine content of SF-WS was quantified by Ellman’s assay. Briefly, SF-WS were dissolved in 0.1 M sodium phosphate buffer at various concentrations and 200 µL was added to a 96-well plate. DTNB reagent was dissolved in 0.1 M sodium phosphate buffer (pH 8.0) at a concentration of 4 mg/mL. DTNB solution (10 µL) was added to each well, and incubated for 15 min. The absorbance was measured using a microplate reader (Autobio, China). The cysteine content of each sample was estimated by comparing its absorbance with that of standard solutions of cysteine hydrochloride.

The primary amine content of samples was quantified by fluorescamine assay. SF-WS and SF-EGCG were dissolved in purified water at various concentrations (0.125, 0.25, 0.5, 1 mg/mL), and 150 µL was added to a 96-well plate. Fluorescamine reagent was dissolved in acetone at a concentration of 3 mg/mL. The fluorescamine solution (50 µL) was added to each well, and the plate was shaken on a plate shaker for 1 min. Next, the fluorescence intensity was measured by Synergy HT ELISA reader (BioTek, USA) with an excitation wavelength at 390 nm and an emission wavelength at 515 nm. The primary amine content of each sample was estimated by comparing its fluorescence intensity with that of glycine standard solutions. DS was determined based on the moles of substituted amino acids using the following equation:

$$DS = {\text{ }}\frac{{Moles{\text{ }}of{\text{ }}substituted{\text{ }}amino{\text{ }}acids{\text{ }}per{\text{ }}gram{\text{ }}of{\text{ }}sample}}{{Moles{\text{ }}of{\text{ }}amino{\text{ }}acid{\text{ }}residues{\text{ }}per{\text{ }}gram{\text{ }}of{\text{ }}sample}} \times 100$$

, where the number of moles of amino acid residues was calculated based on the average molecular mass (*M*_a_) of an amino acid residue in silk fibroin.

$${M_a} = \frac{{\sum {N_i}\left( {{M_i} - {M_{water}}} \right)}}{{\sum {N_i}}}$$

, where *M*_water_ is a molar mass of water (18 g/mol) and *N*_i_ is the number of each amino acid with a molar mass *M*_i_. The amino acid composition of silk fibroin was referred from a previous study [9].

### Evaluation of ROS scavenging and collagenase-inhibitory activity

Hydroxyl radical (•OH) scavenging activity was determined by the deoxyribose method [33]. Briefly, 500 µM FeSO_4_, 20 mM 2-deoxy-D-ribose, and 500 µM ascorbic acid were prepared in 0.1 M sodium phosphate buffer. Separately, 5 mM H_2_O_2_, 28 mg/mL of trichloroacetic acid, and various concentrations of sample (SF-WS, SF-T, and SF-EGCG) solutions were prepared in purified water. Thiobarbituric acid (10 mg/mL) was prepared in a 50 mM NaOH solution. FeSO_4_, 2-deoxy-D-ribose, ascorbic acid, H_2_O_2_ and sample solutions were mixed in 0.5-mL polymerase chain reaction (PCR) strip as 50 µL. After the mixed solution was incubated for 1 h at 37°C, thiobarbituric acid and trichloroacetic acid solution was added and then heated for 15 min at 99°C. Afterwards, the solution was cooled at room temperature, and then transferred to a 96-well plate (150 µL). The absorbance at 540 nm was observed by a microplate reader (Autobio, China). Hydroxyl radical scavenging activity was calculated by the following equation:

$$Hydroxyl{\text{ }}radical{\text{ }}scavenging{\text{ }}activity\left( \%  \right) = {\text{ }}\frac{{{A_{control}} - {A_{sample}}}}{{{A_{control}}}} \times 100$$

, where A_control_ and A_sample_ represent the absorbance of control and sample, respectively. For the control experiment, 0.1 M sodium phosphate buffer was used.

Superoxide radical (O_2_•¯) scavenging activity was determined by oxidation of xanthine [34]. Xanthine oxidase (1 unit/mL) and 4-nitro blue tetrazolium chloride (NBT, 0.1 mM) were prepared in 0.1 M sodium phosphate buffer. Samples (SF-WS, SF-T, SF-EGCG) were dissolved in purified water at varying concentrations. Xanthine (Daejung Chemical and Metals, Korea) was dissolved in 1 M NaOH solution at a concentration of 1 mM, and diluted 5-fold with purified water before use. The xanthine solution (60 µL), NBT solution (60 µL), xanthine oxidase (40 µL) and sample solution (40 µL) were mixed in a 96-well plate, and then incubated for 15 min (37°C, dark place). Afterwards, the absorbance at 560 nm was observed by a microplate reader (Autobio, China). Superoxide radical scavenging activity was calculated by the following equation:

$$Superoxide{\text{ }}radical{\text{ }}scavenging{\text{ }}activity\left( \%  \right) = {\text{ }}\frac{{{A_{control}} - {A_{sample}}}}{{{A_{control}}}} \times 100$$

, where A_control_ and A_sample_ represent the absorbance of control and sample, respectively. For the control experiment, purified water was used. Collagenase-inhibitory activity of SF-WS and SF-T were determined using Enzchek^®^ gelatinase/collagenase assay kit according to the manufacturer’s instruction.

### Cytocompatibility assay

Cytocompatibility of SF-WS and SF-EGCG was evaluated using the MTT method. NIH3T3 fibroblasts (Korea Cell Line Bank, Korea) were maintained in Dulbecco’s Modified Eagles medium (DMEM) supplemented with 10% (v/v) fetal bovine serum. The cells were seeded in a 96-well plate at a density of 10^4^ cells/well and then cultured for 24 h (37°C, 5% CO_2_). After the medium was removed, the cells were treated with various concentrations of SF-WS and SF-EGCG for 24 h at 37°C. After rinsing with PBS solution, the cells were treated with 100 µL of 3-(4,5-dimethylthiazol-2-yl)-2,5-diphenyltetrazolium bromide (MTT) reagent for 4 h at 37°C. After MTT reagent was removed out, 100 µL of dimethyl sulfoxide was added to each well. After 30 min, the absorbance at 540 nm was measured using a microplate reader (Autobio, China).

### Preparation of SF-T and SF-T/SF-EGCG hydrogels

The SF-T and SF-T/SF-EGCG hydrogels were prepared through HRP-catalyzed enzymatic crosslinking reaction. SF-T and SF-EGCG conjugates were dissolved in PBS solution (pH 7.4) at a concentration of 10 wt%. HRP was dissolved in PBS solution at a concentration of 20 unit/mL, and H_2_O_2_ (30%) was diluted to 100 mM. The final polymer concentration was 5 wt%, and the weight ratio between SF-T and SF-EGCG was adjusted to 100:0, 90:10, 70:30, and 50:50, respectively. All components (Table 1) were mixed in a 1.5-mL conical tube, poured into a disc-shaped silicone mold (20 mm diameter × 3 mm height) and then incubated in a humid chamber for 2 h at 37°C for crosslinking reaction.

**Table 1:** Optimized compositions of SF-T and SF-T/SF-EGCG composite hydrogels on storage modulus and gelation time for wound dressing application

Hydrogels	SF-T (wt%)	SF-EGCG (wt%)	HRP (unit/mL)	H_2_O_2_ (mM)
SF-T	5.0	0	0.7	5.0
SF-T90/SF-EGCG10	4.5	0.5	0.8	6.4
SF-T70/SF-EGCG30	3.5	1.5	1.2	10.5
SF-T50/SF-EGCG50	2.5	2.5	3.5	13.5

### Evaluation of storage modulus, gelation time and microstructure of hydrogels

SF-T and SF-T/SF-EGCG hydrogels were placed between the parallel plates (20 mm in diameter) of a rotational rheometer (ARES, TA Instruments, USA) with a gap of 2 mm for frequency sweep test. The storage modulus (*G*`) was measured between 0.2 and 10 Hz under a constant strain (1%) at 25°C. The gelation time of the hydrogels was measured by the vial tilting method. Briefly, the pre-gel solution (0.5 g) was prepared in a 1.5-mL conical tube and vortexed for 2 s. Afterwards, the tube was tilted every 1 s, and the time was recorded when the pre-gel solution stopped flowing. The microstructure of the lyophilized hydrogels was observed using a scanning electron microscope (SEM, JSM-6380, JEOL, Japan) at an accelerating voltage of 10–20 kV. The surface of the construct was coated with platinum by a sputter coater before observation.

### ***In vitro*** swelling and stability studies

SF-T and SF-T/SF-EGCG hydrogels were fabricated in circular shaped mold (20 mm diameter × 3mm height). To examine the equilibrium swelling ratio, lyophilized hydrogels were immersed in PBS solution (pH 7.4) at 37ºC for 24 h and then weighed after wiping off excess water. The equilibrium swelling ratio was calculated by dividing the mass of swollen gel with the mass of dried gel. For in vitro stability studies, the specimens were inserted into conical tubes containing 50 mL PBS solution and incubated for 21 days at 37°C. Stability of the hydrogels was assessed by measuring their weight loss using the following equation:

$$Weight{\text{ }}loss\left( \%  \right) = {\text{ }}\frac{{{W_i} - {W_d}}}{{{W_i}}} \times 100$$

, where W_i_ and W_d_ represent the initial weight of hydrogels and the weight at a designated time point, respectively. At selected time points (1 and 9 days), the hydrogels were retrieved from PBS solution and then examined using attenuated total reflection-Fourier transform infrared (ATR-FTIR) spectrometer (Nicolet 6700, Thermo Fisher Scientific, USA). The amide I band of fibroin β-sheet (1624 cm^− 1^) and the amide I band of fibroin random coil (1650 cm^− 1^) were assessed [35].

### ***In vivo*** wound healing test

The wound healing experiment with the full thickness skin defect model was conducted using male Sprague Dawley rats (240 ~ 250 g, 7 ~ 8 weeks old, Hyochang science, Korea). All animal experiments were reviewed and approved by the Institutional Animal Care and Use Ethics Committee of the Kyungpook National University. Experimental procedures were approved by the Animal Care Committee (2017 − 0131). All rats were divided into 5 groups randomly and acclimatized for 5 days before surgery. The rats were anesthetized by laparoscopic injection of 2,2,2-tribromoethanol (Avertin, 240 mg/kg body weight, Sigma Aldrich, USA). A full thickness skin defect (2 cm in diameter) was created in dorsal region. The wounds treated with a cotton gauze (Daehan Medical, Korea) served as a negative control. SF-T, SF-T70/SF-EGCG30, SF-T50/SF-EGCG50 hydrogels were applied to the wounds as experimental groups. For comparison, a commercial hydrogel dressing (DuoDERM^®^ hydroactive^®^ gel, ConvaTec, USA) was tested. After application of the hydrogels onto the wound area, Tegaderm™ film dressing (3 M, USA) was used to avoid detachment of the hydrogel dressings. The wound size was recorded and the wound dressings were replaced on days 1, 3, 5, 7, 10, and 14. The wound size was calculated using an image analyzer (I-solution lite, IMT i-solution, Korea). For histological evaluation, tissue specimen in the wound area was collected and fixed for 24 h in 10% formalin solution. The fixed tissue was embedded in paraffin and then cross-sectioned to 5 μm thickness. The sections were deparaffinized and stained with hematoxylin-eosin (H&E) and Masson’s trichrome. Each slide was mounted and observed using an optical microscope (ECLIPSE TS100, Nikon, Japan) equipped with a digital camera (DS-Fi-2, Nikon, Japan). The quantitative area of inflammatory cells and collagen deposition was calculated using an image analyzer (ImageJ, 1.53e, USA).

### Statistical analysis

Data are expressed as mean ± standard deviation (SD) unless otherwise stated. All data were analyzed by Shapiro-wilk test to confirm the normal distribution. Statistical comparisons between groups were performed by matched pair t-test and one-way ANOVA with Tukey’s multiple comparison test using the Kyplot software (KyensLab, Japan). Statistical significance was considered with *p* values less than 0.05.

## Results

### Characterization of SF-EGCG conjugate

Natural SF fibers contain crystalline regions mainly composed of β-sheet structure and thus require a regeneration process to obtain an amorphous random-coil conformation with high water solubility for biomedical use [36]. However, regenerated SF tends to reform β-sheet structure and aggregate into water-insoluble fibrils upon lyophilization. To circumvent this limitation, we carried out thermal hydrolysis of SF. It was confirmed through GPC that the molecular weight of the SF prepared through heat treatment was reduced. Water-soluble SF derivative (SF-WS) was prepared by thermal hydrolysis of SF over 7 h at 100ºC to produce short chain [31]. Gel permeation chromatography (Table S1) revealed the weight-average molecular weight (*M*_w_) of SF-WS (15.6 kDa) was markedly smaller than those of native SF (up to 500 kDa) [37]. The regenerated SF-WS powder was fully soluble in water without β-sheet formation, and thus used for all subsequent experiments.

**Fig. 1:**
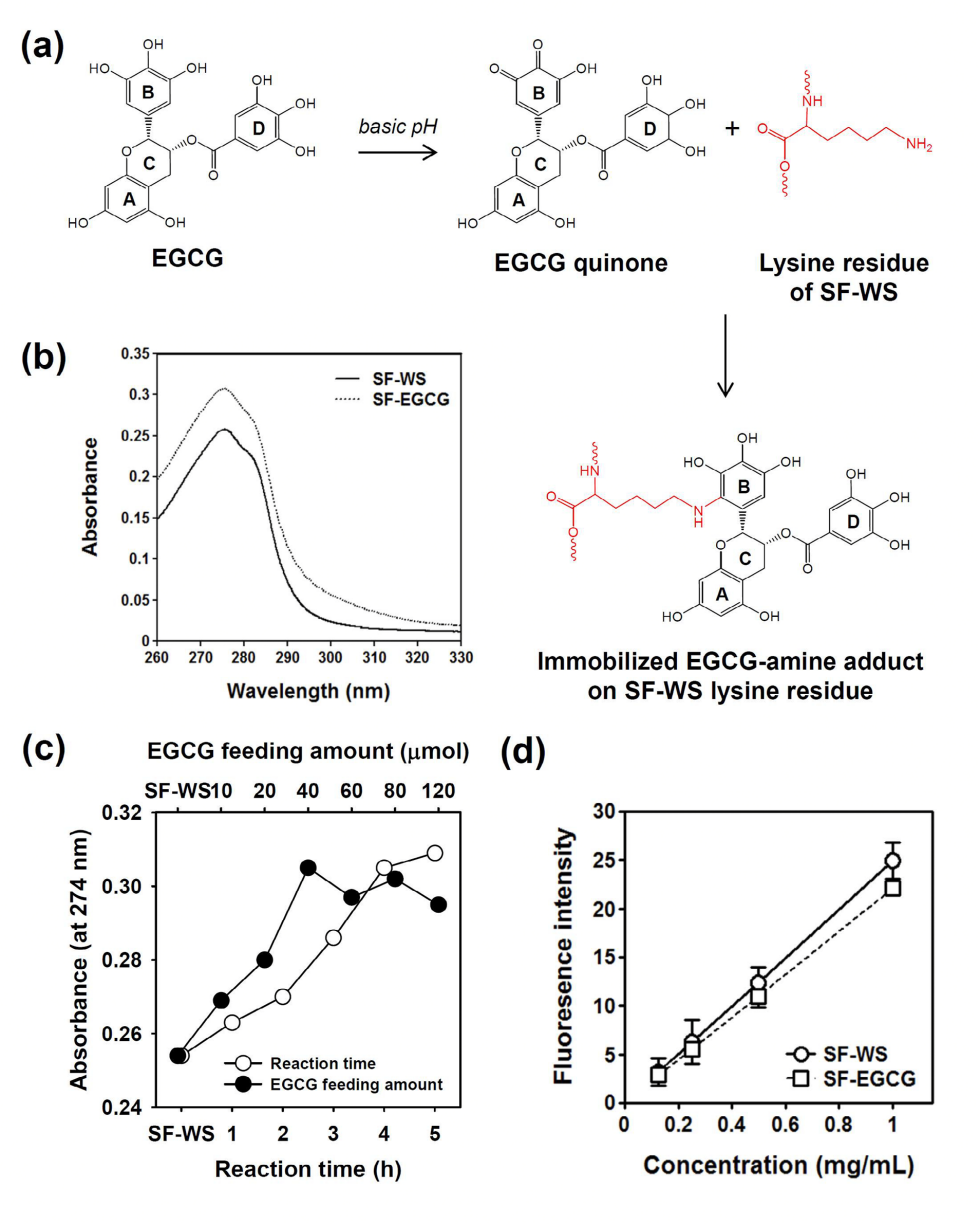
(a) Scheme of the SF-EGCG conjugate formation through autoxidation of EGCG at basic pH 7.4 and subsequent conjugation reaction of EGCG quinone with a lysine residue of SF-WS. (b) UV-visible spectra of SF-WS and SF-EGCG solutions at an equal concentration (50 µg/mL). (c) Optimization of EGCG feeding amount and reaction time. Absorbance at 274 nm of SF-EGCG conjugates formed at various reaction time (feeding amount of EGCG = 40 µmol) and various feeding amounts of EGCG (reaction time = 4 h). (d) Fluorescence intensity (λ_ex_ = 390 nm, λ_em_ = 515 nm) of SF-WS and SF-EGCG solutions on primary amine content after 1 min of reaction with fluorescamine assay reagent (*n* = 8)

Figure 1a depicts the synthesis scheme for SF-EGCG conjugate having multiple EGCG moieties along the SF-WS backbone. Under mild alkaline condition (pH 7.4), EGCG is autoxidized to form an *ortho*-quinone selectively at the pyrogallol moiety (B ring) [38]. The nucleophilic addition of a lysine residue of SF-WS to the *ortho*-quinone moiety leads to the formation of amine-quinone adduct at the B ring of EGCG. Of note, amine-quinone adduct is considered the dominant final product as the formation of EGCG-quinone imine is unfavorable due to rapid hydrolysis of the imine bond in aqueous solution (Fig. S1). The resulting SF-EGCG conjugate was purified by extensive dialysis using nitrogen-purged water and then lyophilized to obtain a dry product.

UV-visible spectrum of SF-EGCG conjugate showed much larger absorption peak at 274 nm than SF-WS, demonstrating the conjugation of EGCG moieties on SF-WS (Fig. 1b). To optimize the reaction time, EGCG conjugation reaction was carried out for various periods of time (1 ~ 5 h). UV-visible spectroscopy of the products revealed that absorbance at 274 nm gradually increased over 4 h, indicating time-dependence of EGCG conjugation reaction (Fig. 1c). Since a further incubation up to 5 h did not increase the absorbance significantly, the optimal reaction time was determined as 4 h. To optimize the feeding amount of EGCG, various quantities (10 ~ 80 µmol) of EGCG were mixed with 1 g of SF-WS before proceeding the reaction for 4 h. The absorbance at 274 nm was the largest when the feeding amount of EGCG was 40 µmol. Raising the feeding amount of EGCG up to 80 µmol did not help to increase the absorbance, suggesting that the reaction reached a saturation state above 40 µmol of EGCG. Thus, SF-EGCG conjugate produced with 40 µmol of EGCG was used for subsequent studies.

The extent of EGCG conjugation was measured by comparing the difference in absorbance between SF-EGCG and SF-WS with a series of EGCG standards (Fig. S2). The degree of substitution (DS) of SF-EGCG conjugate was determined as 0.14 based on the amount of conjugated EGCG moieties (18.63 µmol/g) and the average molecular mass (${M_a}$) of an amino acid residue in silk fibroin (74.66 g/mol). This DS value is considered reasonable because native SF contains only 0.3 mol% of lysine [39]. Next, we performed fluorescamine assay to examine the extent of lysine side chain modifications [40]. Fluorescamine assay (Fig. 1d) revealed that the amount of reacted lysine residues was 18.50 µmol/g, which was very close to that of conjugated EGCG moieties (18.63 µmol/g). These results demonstrated that EGCG conjugation reactions occurred primarily at lysine residues in SF-WS.

### ROS scavenging and protease inhibitory effects of SF-EGCG conjugate

**Fig. 2:**
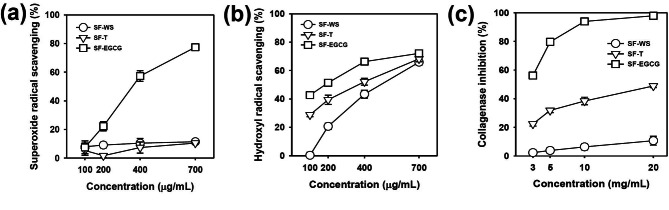
(a) Superoxide anion radical (O_2_•¯) and (b) hydroxyl radical (•OH) scavenging activity (*n* = 8) and (c) collagenase inhibitory activity (*n* = 6) of SF-WS, SF-T and SF-EGCG as a function of concentration

We examined the ability of SF-EGCG conjugate to scavenge two different types of ROS: superoxide anion radical (O_2_•¯) and hydroxyl radical (•OH) which are highly deleterious ROS abundant in the wound environment [7]. For comparison, SF-T conjugate with DS of 1.37 was synthesized by a carbodiimide coupling reaction (Fig. S3) according to the previous report [27]. SF-EGCG conjugates induced significant, dose-dependent scavenging effects on O_2_•¯, while only a marginal scavenging effect was observed from SF-T and SF-WS (Fig. 2a). In addition, the strongest •OH scavenging activity was observed with SF-EGCG, followed by SF-T and SF-WS (Fig. 2b). SF-T and SF-WS were found to have moderate •OH scavenging effects at concentrations above 400 µg/mL. As increasing SF polymer concentrations, the gap in •OH scavenging activity among the three groups was narrowed by the inherent •OH scavenging activity of SF backbone [41]. It is worth noting that SF-EGCG exhibited greater ROS-scavenging activity than SF-T although DS of SF-EGCG (0.14) was much lower than that of SF-T (1.37). The superior ROS scavenging effect of SF-EGCG conjugates was likely ascribed to the existence of aromatic ring structures in EGCG moiety, which are capable of capturing and neutralizing free radicals [28, 33]. The SF-EGCG conjugate has scavenging efficacy. In addition, we investigated scavenging efficacy of crosslinked SF-T/SF-EGCG composite hydrogels. And, we confirmed the consistent results the SF-T/SF-EGCG composite hydrogels have ROS scavenging efficacy (Fig. S4).

Considerable attention has been paid to EGCG because of its ability to inactivate collagenase in a competitive manner by binding to its catalytic domain [42, 43]. As shown in Fig. 2c, SF-EGCG exerted stronger collagenase-inhibitory activity than SF-T and SF-WS. Even though DS of SF-EGCG (0.14) was almost 10-fold lower than that of SF-T (1.37), SF-EGCG was much more efficient in suppressing the activity of collagenase than SF-T. EGCG has been reported to exhibit about 100-fold higher affinity towards human serum albumin than (–)-epicatechin or (–)-epigallocatechin, which lacks a galloyl moiety, suggesting that the galloyl moiety plays a crucial role in EGCG-protein interactions *via* hydrogen-bonding and hydrophobic forces [42 − 44]. Hence, it is conceivable that SF-EGCG could bind to collagenase more strongly than SF-T, leading to more effective inhibition of its enzymatic activity.

### Formation of SF-T/SF-EGCG hydrogels

**Fig. 3:**
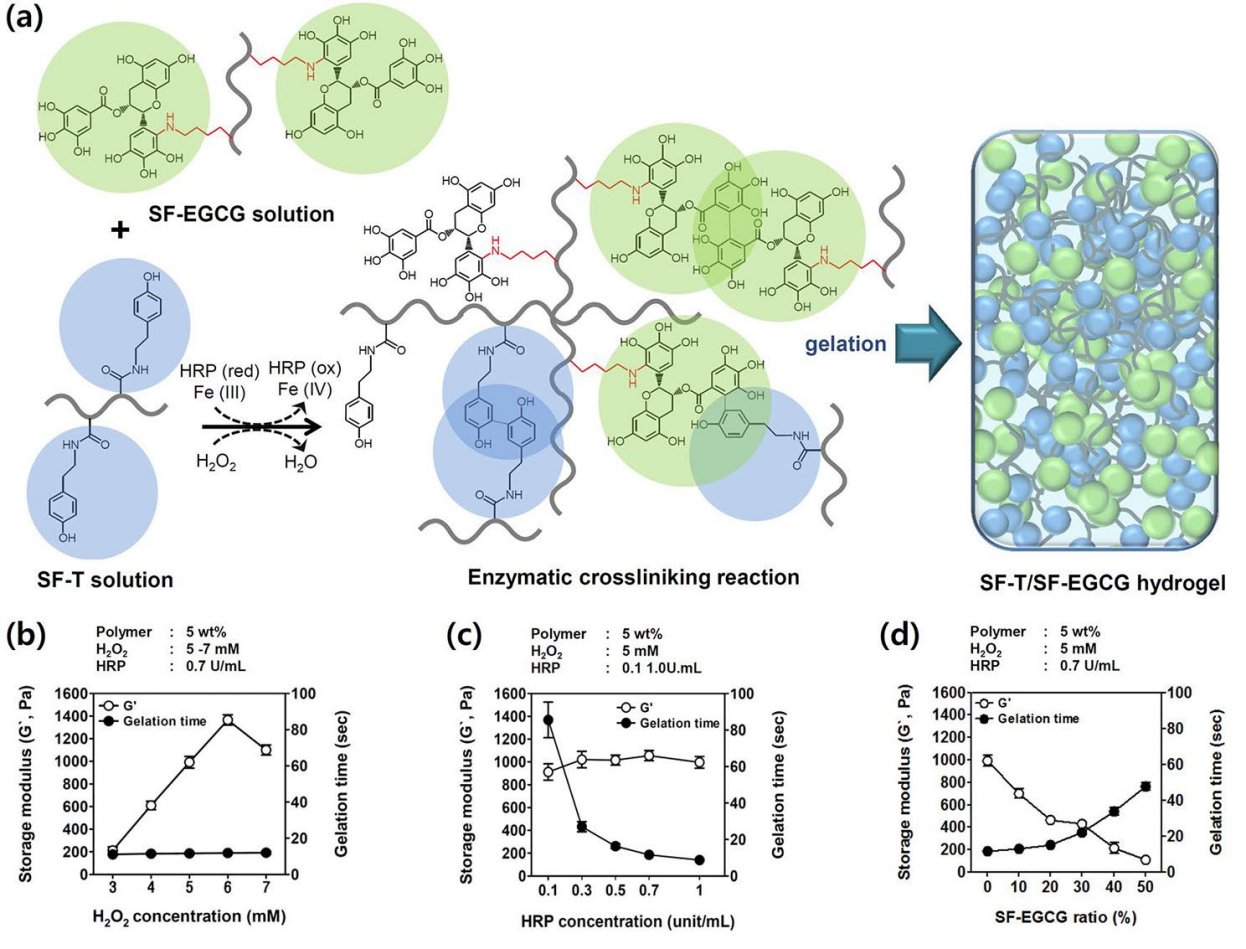
(a) Schematic for the chemical formation of SF-T/SF-EGCG composite hydrogels *via* HRP/H_2_O_2_-mediated enzymatic crosslinking reaction. (b) Storage modulus and gelation time of SF-T hydrogels as a function of H_2_O_2_ concentration. The concentration of HRP was fixed to 0.7 unit/mL. (c) Storage modulus and gelation time of SF-T hydrogels as a function of HRP concentration. The concentration of H_2_O_2_ was fixed to 5 mM. (d) Effect of the addition of SF-EGCG on the storage modulus and gelation time of SF-T/SF-EGCG composite hydrogels. The concentration of H_2_O_2_ and HRP was fixed to 5 mM and 0.7 unit/mL, respectively. (Mean ± SD, *n* = 5)

Motivated by the above findings, we have fabricated EGCG-grafted SF-based composite hydrogels through co-crosslinking SF-EGCG and SF-T using HRP/H_2_O_2_-mediated enzymatic reaction (Fig. 3a). It has been reported that a catalytic cycle of HRP converts phenolic moieties of tyramine and EGCG to phenoxy free radicals while consuming H_2_O_2_ and releasing water molecules as a byproduct [32, 45]. The phenoxy free radicals can react with each other to produce cross-linkages between SF-EGCG and SF-T conjugates.

First, we attempted to fabricate the SF-T hydrogel without SF-EGCG (Fig. 3b and c). As reported in the previous report, high concentrations of H_2_O_2_ increased the storage modulus of hydrogel but denatured HRP over excessive concentration [46]. Also, the increase of HRP concentration highly shortened gelation time without affecting the mechanical strength [47]. Next, we investigated the effect of the addition of SF-EGCG on the storage modulus and gelation time of SF-T/SF-EGCG composite hydrogels. Generally, the mechanical properties of hydrogels should be matched with those of native skin tissue to promote the restoration of defected wound area. In addition, gelation time should be optimized to < 30 s to achieve quick coverage of skin defects as well as to minimize the diffusion of the gel precursors away from the wound site. Hence, the concentration of H_2_O_2_ and HRP was set to 5 mM and 0.7 unit/mL, respectively, because SF-T hydrogels formed under this condition exhibited rapid in situ gelation (~ 10 s) and *G*` value (~ 1000 Pa) similar to those of human skin [48]. It is reported that the wound closure and growth factor secretion are improved under the proper storage modulus of about 1000 Pa which is sufficiently robust to support but doesn’t hinder the wound contraction [49]. At the fixed concentration of H_2_O_2_ and HRP, SF-T/SF-EGCG composite hydrogels were produced by varying the weight ratio of SF-T to SF-EGCG from 90:10 to 50:50. Interestingly, raising the ratio of SF-EGCG gradually decreased *G*` of SF-T/SF-EGCG hydrogels with a concomitant increase in gelation time (Fig. 3d). This finding can be explained by the scavenging of phenoxy free radicals by greater amounts of EGCG moieties, leading to an inhibition in the crosslinking reaction [39].

### Mechanical and physical behavior of hydrogels

**Fig. 4:**
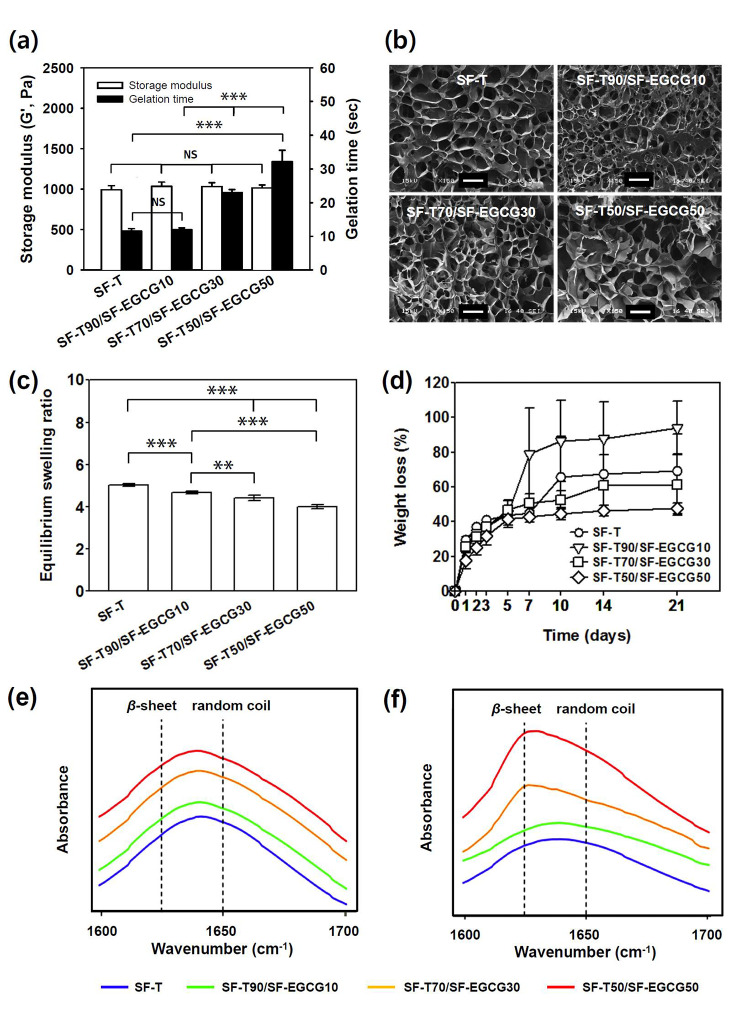
(a) Storage modulus and gelation time of the optimized SF-T hydrogel and SF-T/SF-EGCG composite hydrogels. (Mean ± SD, *n* = 5, NS: not significant, ^***^: *p* < 0.001). (b) Photomicrographs of cross-section of the lyophilized SF-T and SF-T/SF-EGCG composite hydrogels. Scale bar = 100 μm. (c) Equilibrium swelling ratio of the SF-T and SF-T/SF-EGCG composite hydrogels incubated in PBS for 24 h and (d) their weight loss profile in PBS over 21 days. (Mean ± SD, *n* = 7, ^**^: *p* < 0.01, ^***^: *p* < 0.001). ATR-FTIR spectra of the hydrogels on (e) day 1 and (f) day 9. The dashed lines indicate the amide I band of fibroin β-sheet (1624 cm^− 1^) and the amide I band of fibroin random coil (1650 cm^− 1^)

As presented in Fig. 4a, all optimized SF-T and SF-T/SF-EGCG hydrogels had desirable *G*` values (ca. 1,000 Pa) and sufficiently rapid gelation time (up to ~ 32 s). SEM images revealed the presence of interconnected microscopic pores within the optimized hydrogels (Fig. 4b), which is advantageous to promote tissue perfusion and oxygenation for wound healing applications.

The equilibrium swelling ratios of SF-T/SF-EGCG composite hydrogels were much lower than those of SF-T hydrogels (Fig. 4c). Increasing the mixing ratio of SF-EGCG caused a gradual decrease in the swelling ratios of SF-T/SF-EGCG hydrogels, probably due to the higher hydrophobicity of EGCG (log P = 3.08) compared to tyramine (log P = 0.68). Also, the SF-T/SF-EGCG composite hydrogel showed ROS scavenging activity like SF-EGCG conjugate (Fig. S4). In vitro stability of SF hydrogels was assessed by measuring their weight loss in PBS solution (pH 7.4) at 37ºC. SF-T hydrogels showed poor stability in the physiological environment with a loss of about 70% of the initial weight over 21 days (Fig. 4d). A faster weight loss was observed from SF-T90/SF-EGCG10 hydrogels, while SF-T70/SF-EGCG30 and SF-T50/SF-EGCG50 hydrogels showed markedly delayed weight loss. Interestingly, SF-T50/SF-EGCG50 hydrogels were more stable than SF-T70/SF-EGCG30 hydrogels, implying that EGCG content plays an important role in enhancing the stability of SF-T/SF-EGCG composite hydrogels. To understand the reason for the stabilization, ATR-FTIR was conducted to monitor changes in the β-sheet content in the hydrogels. There was only negligible change in the β-sheet content of SF and SF-T90/SF-EGCG10 hydrogels during incubation in PBS solution for 9 days (Fig. 4e and f). On the other hand, a drastic increase in the β-sheet content was observed from SF-T70/SF-EGCG30 and SF-T50/SF-EGCG50 hydrogels. It has been reported that crosslinking of the tyrosine residues in silk fibroin induced the formation of extended β-sheet structure through π − π interactions and hydrogen bonding with adjacent peptide backbone [35]. Considering the ability of EGCG moieties to form π − π interactions and hydrogen bonding [28], the increased amount of SF-EGCG within SF-T70/SF-EGCG30 and SF-T50/SF-EGCG50 hydrogels was likely responsible for the enhanced β-sheet formation and delayed dissociation of silk fibroin chains. Given the excellent stability, SF-T70/SF-EGCG30 and SF-T50/SF-EGCG50 hydrogels were chosen for the rest of the studies.

### ***In vivo*** wound healing effect

**Fig. 5:**
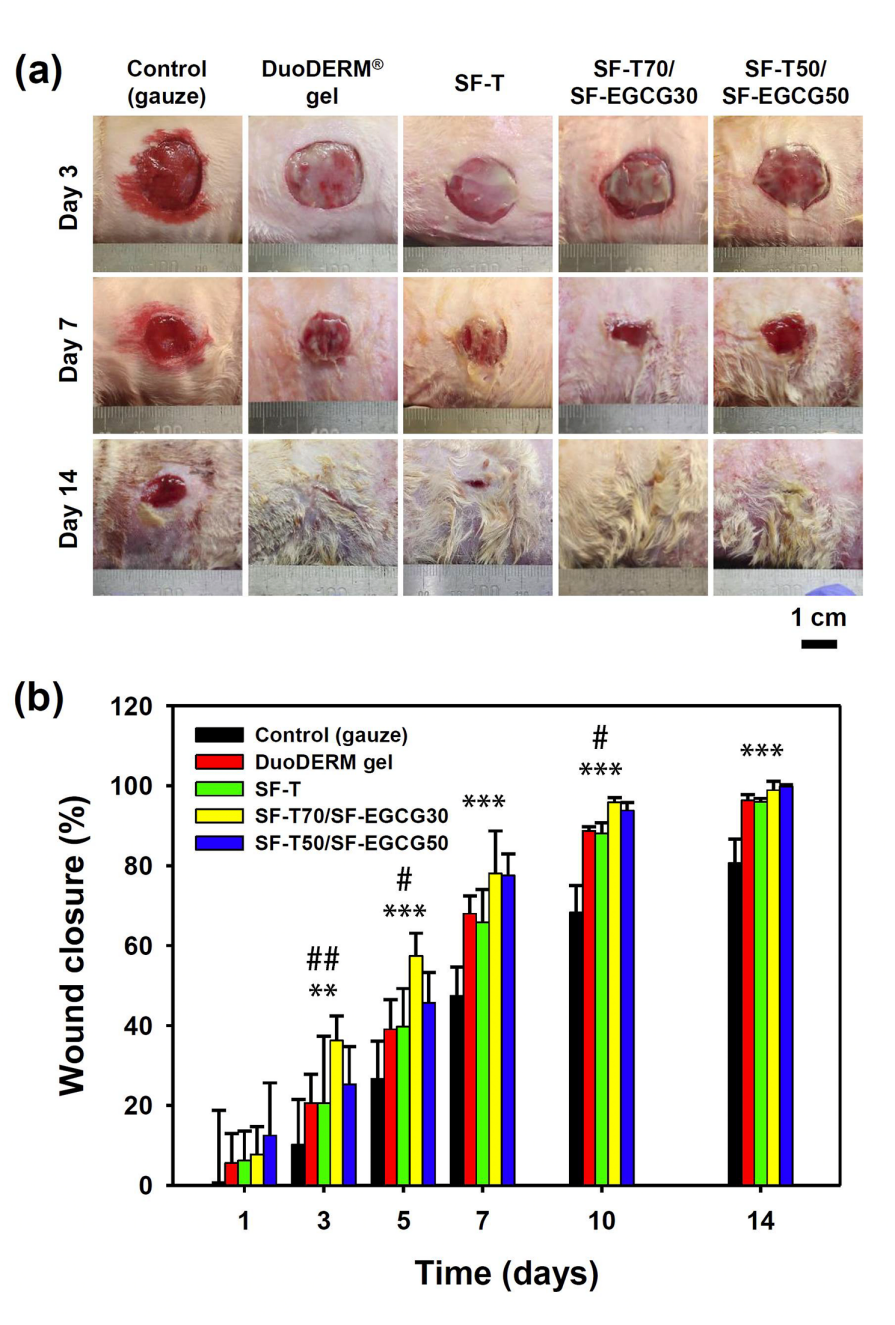
(a) Representative photographs of the wound area and (b) wound closure percentage treated with a gauze (control), DuoDERM® gel, SF-T and SF-T/SF-EGCG hydrogels for 14 days after surgery. (Mean ± SD, *n* = 8, ^**^: *p* < 0.01 and ^***^: *p* < 0.001 for SF-T70/SF-EGCG30 versus the control, ^#^: *p* < 0.05 and ^##^: *p* < 0.01 for SF-T70/SF-EGCG30 versus SF-T).

Wound healing efficiency of SF-T/SF-EGCG composite hydrogels was evaluated in a rat model of full thickness skin defect. After the wounds were created, SF-T/SF-EGCG hydrogels, SF-T hydrogel, cotton gauze (control) and DuoDERM® gel (a commercial hydrogel dressing) were applied to cover the wounds and the recovery process was monitored at different time points. The wounds treated with the cotton gauze failed to heal completely even after 14 days (Fig. 5a). In addition, bleeding was consistently observed from the gauze group on day 3 and 7. Since the cotton gauze is a dry dressing and unable to keep the wounds moisture, strong adhesion between the gauze and wound bed usually occurs.

Quantification of the wound closure showed that SF-T70/SF-EGCG30 and SF-T50/SF-EGCG50 hydrogels facilitated wound closure more quickly than the other groups (Fig. 5b). For instance, the order of effectiveness on day 7 was SF-T70/SF-EGCG30 ≈ SF-T50/SF-EGCG50 > SF-T ≈ DuoDERM® gel > gauze control. Notably, the SF-T/SF-EGCG hydrogels were more effective in promoting wound recovery than the SF-T hydrogel and DuoDERM® gel lacking EGCG moieties. It suggests that the superior ROS scavenging activity of SF-EGCG probably contributed to the accelerated healing effect of the SF-T/SF-EGCG hydrogels.

**Fig. 6:**
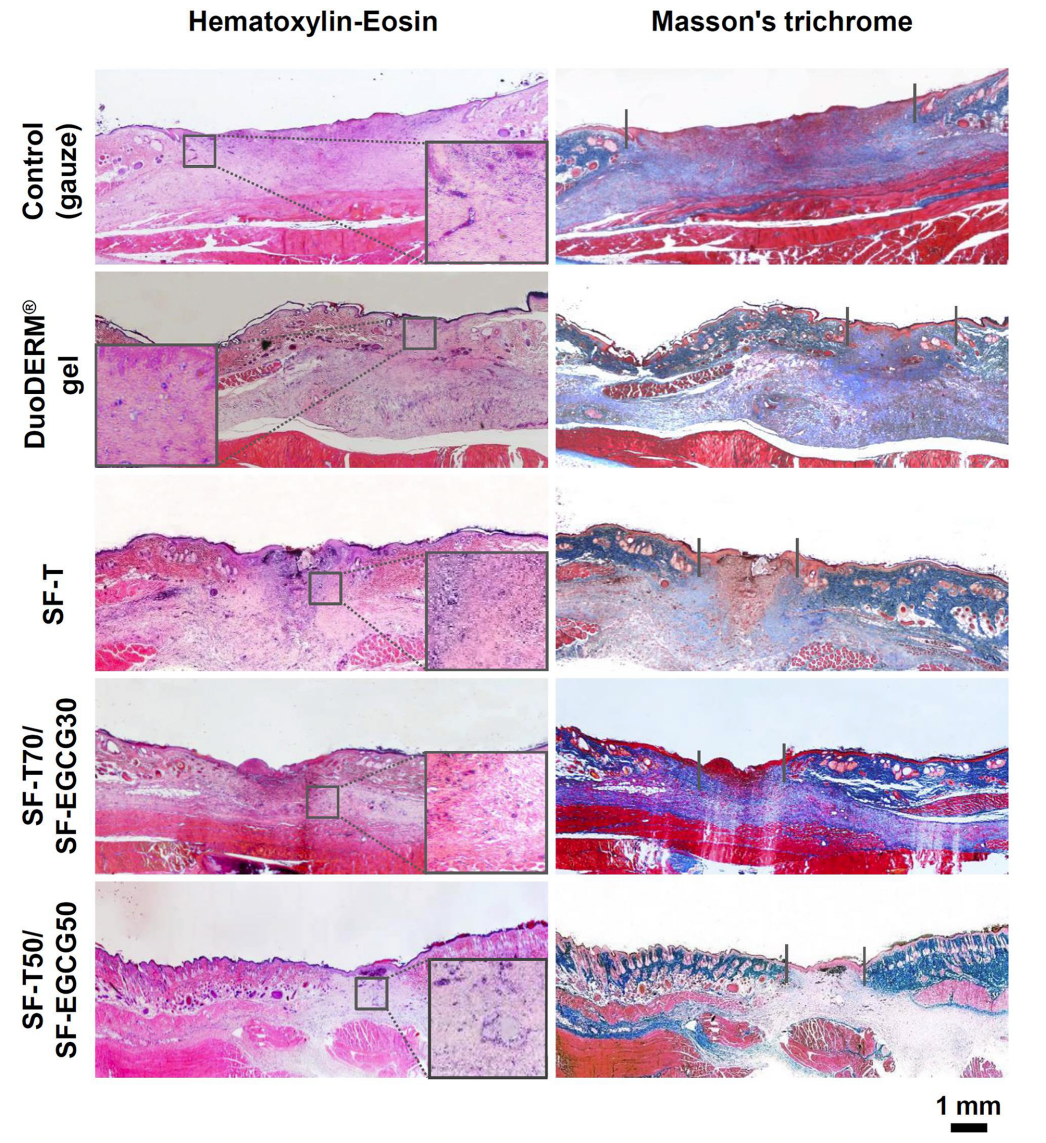
Histological photomicrographs of the wound area harvested after 14 days of treatments with a gauze (control), DuoDERM® gel, SF-T and SF-T/SF-EGCG hydrogels. The left and right panels show hematoxylin-eosin (H&E) staining (pink; cytoplasm, blue purple; nuclei) and Masson’s trichrome staining (pink; cytoplasm, blue; collagen and connective tissues, dark red; keratin and muscle fibers, blue purple; nuclei), respectively. The insets represent inflammatory cells (macrophages, lymphocytes, neutrophils, eosinophils) at the interface between matured and healed wound area

**Fig. 7:**
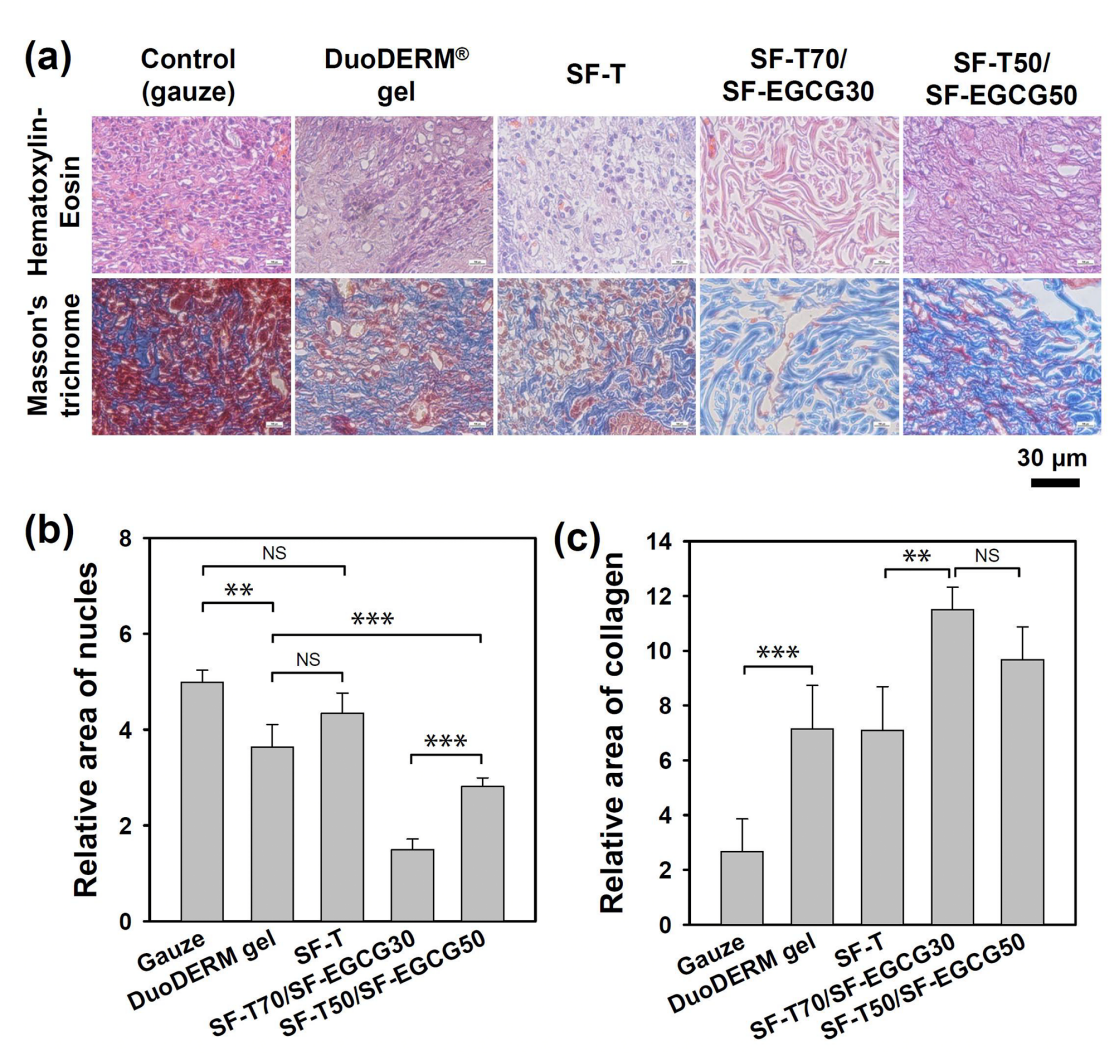
(a) High resolution histological microphotographs of regenerated wound area after 14 days of treatments with a gauze (control), DuoDERM® gel, SF-T and, SF-T/SF-EGCG hydrogels. Quantitative image analysis of (b) inflammatory cells and (c) collagen deposition area in the regenerated wound area of each group (Mean ± SD, n = 5, NS: not significant, **: *p* < 0.01, ***: *P* < 0.001)

H&E and Masson’s trichrome staining was employed to assess the distribution of inflammatory cells and regeneration of dermal tissue in the wound area after 14 days of different treatments. The gauze group exhibited incomplete re-epithelialization and significant appearance of inflammatory cells, such as macrophages, lymphocytes and neutrophils (Fig. 6, inset). Both DuoDERM® gel and SF-T groups showed the formation of discontinuous epidermis and irregular dermis tissues although they achieved over 95% of wound closure on day 14. It means that typical hydrogel dressings with a moisture maintaining ability were not sufficient to promote effective wound healing. More complete regeneration of the dermis and epidermis layer was observed from SF-T70/SF-EGCG30 and SF-T50/SF-EGCG50 groups, which was consistent with the wound closing behavior. In addition, maturated collagen tissues (blue), keratin and muscle fibers (deep purple) were obviously observed from SF-T70/SF-EGCG30 (Fig. 7). Both groups contained a far smaller number of inflammatory cells compared to SF-T group. It suggests that SF-T/SF-EGCG hydrogels effectively diminished ROS mediated inflammation probably due to the stronger ROS scavenging activity of SF-EGCG. Cytocompatibility experiments revealed that both SF and SF-EGCG were totally non-toxic to NIH3T3 fibroblasts, indicating that the hydrogel components are not likely to cause skin cell death even if they are released as a result of potential degradation (Fig. S6). The tropical tissue damage and necrosis have not observed at the application site of SF-T70/SF-EGCG30 and SF-T50/SF-EGCG50 hydrogels, indicating that they did not cause any potential side effects during 14 days of usage. In summary, these results demonstrated superior wound healing performance of SF-T/SF-EGCG hydrogels over SF-T hydrogels and even the commercial DuoDERM® gel dressing in the in vivo full-thickness skin defect model.

## Discussion

The first agenda of this study was fabricating a silk fibrin based hydrogel with secured quality. To achieve this goal, we have taken the water-soluble SF (SF-WS) by thermal treatment [31]. The thermal treated SF-WS powder was fully soluble. Because the Mw of SF-WS was significantly reduced from500 kDa to 15.6 kDa [36].

As mentioned before, the EGCG has beneficial function for wound dressing due to its strong ROS scavenging and anti-inflammatory activities. The synthesis of SF-EGCG conjugate was successfully achieved. To prove our hypothesis, the chemistry between the free amine group and EGCG was observed. L-lysine and ethanolamine were reacted with EGCG in a similar condition to SF-EGCG synthesis, and mass spectrometry was measured (Fig. S2). Near the 459 m/z and 499 m/z, EGCG and EGCG-acetonitrile solvent adduct was observed, respectively. Near the 603 m/z and 518 m/z, lysine-EGCG adduct and ethanolamine-EGCG adduct were observed, respectively. However, EGCG-quinone imine was not observed at all (Lysine-EGCG imine = 584.55 and ethanolamine imine = 500.44). Also, EGCG and amine group compounds were bounded with a ratio of 1:1. It means the amine compound and EGCG were not bounded by hydrogen bonds. Therefore, we proved that EGCG and the free amine group reacted by Michael’s addition, and amine-quinone is formed.

Elevated ROS levels in wounds are known to stimulate dermal fibroblasts to secrete matrix metalloproteinases, predominantly interstitial collagenases, resulting in excessive breakdown of the collagen fibers in extracellular matrix and delayed wound healing [5, 6]. The hydroxyl radical scavenging activity of SF-WS, SF-T, and SF-EGCG shown the obvious difference in the low concentration, but the differences became narrow as the increase of concentration. The silk fibroin has hydroxyl radical scavenging activity already. The hydroxyl radical react with the phenol group, and forms a phenoxy radical. The phenoxy radical react with the other phenoxy radical, hydroxyl radical and superoxide radical, and forms diphenol, catechol, and tyrosine hydroperoxide, respectively. In the low concentration, it seems that the hydroxyl radical scavenging activity is more dominantly affected by the tyramine or EGCG modified to silk fibroin than the tyrosine residue in the silk fibroin, and as the increase of the concentration, the effect of tyrosine residue become higher. Considerable attention has been paid to EGCG because of its ability to inactivate collagenase in a competitive manner by binding to its catalytic domain [42, 43]. As shown in Fig. 2c, SF-EGCG exerted stronger collagenase-inhibitory activity than SF-T and SF-WS. Even though DS of SF-EGCG (0.14) was almost 10-fold lower than that of SF-T (1.37), SF-EGCG was much more efficient in suppressing the activity of collagenase than SF-T. EGCG has been reported to exhibit about 100-fold higher affinity towards human serum albumin than (–)-epicatechin or (–)-epigallocatechin, which lacks a galloyl moiety, suggesting that the galloyl moiety plays a crucial role in EGCG-protein interactions *via* hydrogen-bonding and hydrophobic forces [44]. Hence, it is conceivable that SF-EGCG could bind to collagenase more strongly than SF-T, leading to more effective inhibition of its enzymatic activity. Also, the collagenase inhibitory activity of EGCG was improved after the conjugation with SF. Although the silk fibroin backbone can make steric hindrance, other hydrogen bond sites in SF can bind collagenase.

To produce SF-T/SF-EGCG composite hydrogels with desirable *G*` (≈1000 Pa) and gelation time (< 30 s), we performed a series of optimization experiments to find proper concentration of H_2_O_2_ and HRP (Fig. S5). For instance, due to the scavenging effect of EGCG moieties against phenoxy free radicals, the concentration of H_2_O_2_ (6.4 mM) and HRP (0.8 unit/mL) needed to prepare SF-T90/SF-EGCG10 hydrogels were higher than those used to form SF-T hydrogels (Table 1).

The ability of SF-EGCG conjugate to scavenge two different types of ROS: superoxide anion radical (O_2_•¯) and hydroxyl radical (•OH) are highly deleterious ROS abundant in the wound environment. The SF-EGCG exhibited greater ROS-scavenging activity than SF-T although DS of SF-EGCG (0.14) was much lower than that of SF-T (1.37). The superior ROS scavenging effect of SF-EGCG conjugates was likely ascribed to the existence of aromatic ring structures in EGCG moiety, which are capable of capturing and neutralizing free radicals [28, 33]. After the conjugation, the hydroxyl radical scavenging activity was increased, but the superoxide radical scavenging activity was decreased. It is considered that the conjugation attenuates the activity of EGCG, but inherent hydroxyl radical scavenging activity of silk fibroin supplemented attenuation of activity in the hydroxyl radical scavenging assay (Fig. S7). In addition, the specific collagenase activity was calculated. After the conjugation, the collagenase inhibitory activity was increased. The silk fibroin backbone can make steric hindrance after the conjugation. However, in this work, it is considered that additional hydrogen bond sites in the silk fibroin backbone supplemented the steric hindrance. Also, after the conjugation, once an EGCG is bound to the collagenase, other conjugated EGCG get a chance to bind to the collagenase more frequently. It is a similar mechanism that the polymer-antibody conjugation improves the binding affinity of the antibody.

Wound healing efficiency of SF-T/SF-EGCG composite hydrogels was evaluated in a rat model of full thickness skin defect. Quantification of the wound closure showed that SF-T70/SF-EGCG30 and SF-T50/SF-EGCG50 hydrogels facilitated wound closure more quickly than the other groups (Fig. 5b). For instance, the order of effectiveness on day 7 was SF-T70/SF-EGCG30 ≈ SF-T50/SF-EGCG50 > SF-T ≈ DuoDERM® gel > gauze control. The replacement of the gauze dressing not only causes pain and bleeding, but also delays the wound healing process by removing regenerating skin cells. Neither bleeding nor skin detachment was observed from the wounds treated with all SF hydrogels and DuoDERM® gel. Wound closure profile of all experimental groups except the control group was almost completed after 14 days. Because the efficacy of hydrogel wound dressings is excellent than other type of wound dressings. Therefore, the day 14 observation is not significant different. But, inner part of regenerated tissue showed matured collagen deposition with reduced inflammatory cells. It can be seen that a lot of collagen deposition with reduced inflammatory cells of the SF-T70/SF-EGCG30 group (Fig. 7). It suggests that these hydrogels effectively maintained a moist environment within the wounds.

## Conclusions

In the present study, SF-EGCG conjugate was synthesized for the first time using the nucleophilic addition reaction of lysine residues in silk proteins with EGCG quinone under a mild basic condition. This conjugate exhibited superior ROS-scavenging and collagenase-inhibitory activities than SF-T and native SF. Furthermore, SF-T/SF-EGCG composite hydrogels prepared by HRP/H_2_O_2_-mediated enzymatic reaction achieved fast in situ gelation (< 30 s), human skin-like storage modulus (≈1000 Pa), and greater wound healing effects with reduced inflammation compared to SF-T hydrogels and the commercial DuoDERM® gel dressing in a rat model of full thickness skin defect. With these attractive properties, SF-EGCG-based composite hydrogels hold great promise as functional biomaterials for wound healing applications.

### Electronic supplementary material

Below is the link to the electronic supplementary material.

Supplementary Material 1

## Data Availability

The supplementary data are available online at www.xxxxx.com/xxx/s1.

## Declarations

**Author details**.

^1^Department of Polymer Science and Engineering, Kumoh National Institute of Technology, Gumi, Gyeongbuk 39177, Korea. ^2^Institute of Bioengineering and Bioimaging, 31 Biopolis Way, The Nanos, Singapore 138669, Singapore. ^3^Gastrointestinal surgery, Kyungpook National University Chilgok Hospital, Daegu 41404, Korea. ^4^Department of Surgery, Kyungpook National University School of Medicine, Daegu 41944, Korea.

## Abbreviations

SF: silk fibroin.
SF-WS: water-soluble silk fibroin.
SF-T: tyramine-substituted SF.
EGCG: (–)-Epigallocatechin-3-O-gallate.
ROS: reactive oxygen species.
HRP: horseradish peroxidase.
