# Correction: Green tea catechin-grafted silk fibroin hydrogels with reactive oxygen species scavenging activity for wound healing applications

**DOI:** 10.1186/s40824-023-00438-y

**Published:** 2023-09-29

**Authors:** Gyeongwoo Lee, Young-Gwang Ko, Ki Hyun Bae, Motoichi Kurisawa, Oh Kyoung Kwon, Oh Hyeong Kwon

**Affiliations:** 1 Department of Polymer Science and Engineering, Kumoh National Institute of Technology, Gumi, 39177 Gyeongbuk Korea; 2 Institute of Bioengineering and Bioimaging, 31 Biopolis Way, The Nanos, Singapore, 138669 Singapore; 3 Gastrointestinal surgery, Kyungpook National University Chilgok Hospital, Daegu, 41404 Korea; 4 Department of Surgery, Kyungpook National University School of Medicine, Daegu, 41944 Korea

**Biomaterials Research (2022) 26:62**

10.1186/s40824-022-00304-3

The authors of the original article [1] mistakenly omitted co-first authorship attribution between the first two authors: Gyeongwoo Lee and Young-Gwang Ko. Both authors should be considered co-first authors.

