# Supplementary material for: Green tea catechin-grafted silk fibroin hydrogels with reactive oxygen species scavenging activity for wound healing applications
Source: Biomater Res. 2022 Nov 9;26:62. doi: 10.1186/s40824-022-00304-3 (PMC9648025; doi:10.1186/s40824-022-00304-3)
Supplement: Supplementary file 1 — Supplementary Material 1 [file 40824_2022_304_MOESM1_ESM.docx]

**Supporting Information for**

**Green tea catechin-grafted silk fibroin hydrogels with reactive oxygen species scavenging activity for wound healing applications**

Gyeongwoo Lee^a^, Young-Gwang Ko^a^, Ki Hyun Bae^b^, Motoichi Kurisawa^b^, Oh Kyoung Kwon^c^, Oh Hyeong Kwon^a,*^

*^a^Department of Polymer Science and Engineering, Kumoh National Institute of Technology, Gumi, Gyeongbuk 39177, Korea*

*^b^Institute of Bioengineering and Bioimaging, 31 Biopolis Way, The Nanos, Singapore 138669, Singapore*

*^c^Gastric Cancer Center, Kyungpook National University Chilgok Hospital, Daegu 41404, Korea*

*Corresponding author: Professor Oh Hyeong Kwon

E-mail: ohkwon@kumoh.ac.kr

Tel: +82-54-478-7690

Fax: +82-54-478-7710

**Table S1**

Gel permeation chromatography **(**GPC) analysis of SF-WS

| **Sample** | ***M*_n_ (Da)** | ***M*_w_ (Da)** | **Polydispersity index (PDI)** |
| --- | --- | --- | --- |
| SF-WS | 3,922 | 15,599 | 3.98 |

* Note: Weight-average molecular weight (Mw) of native SF is up to 500 kDa.^37^


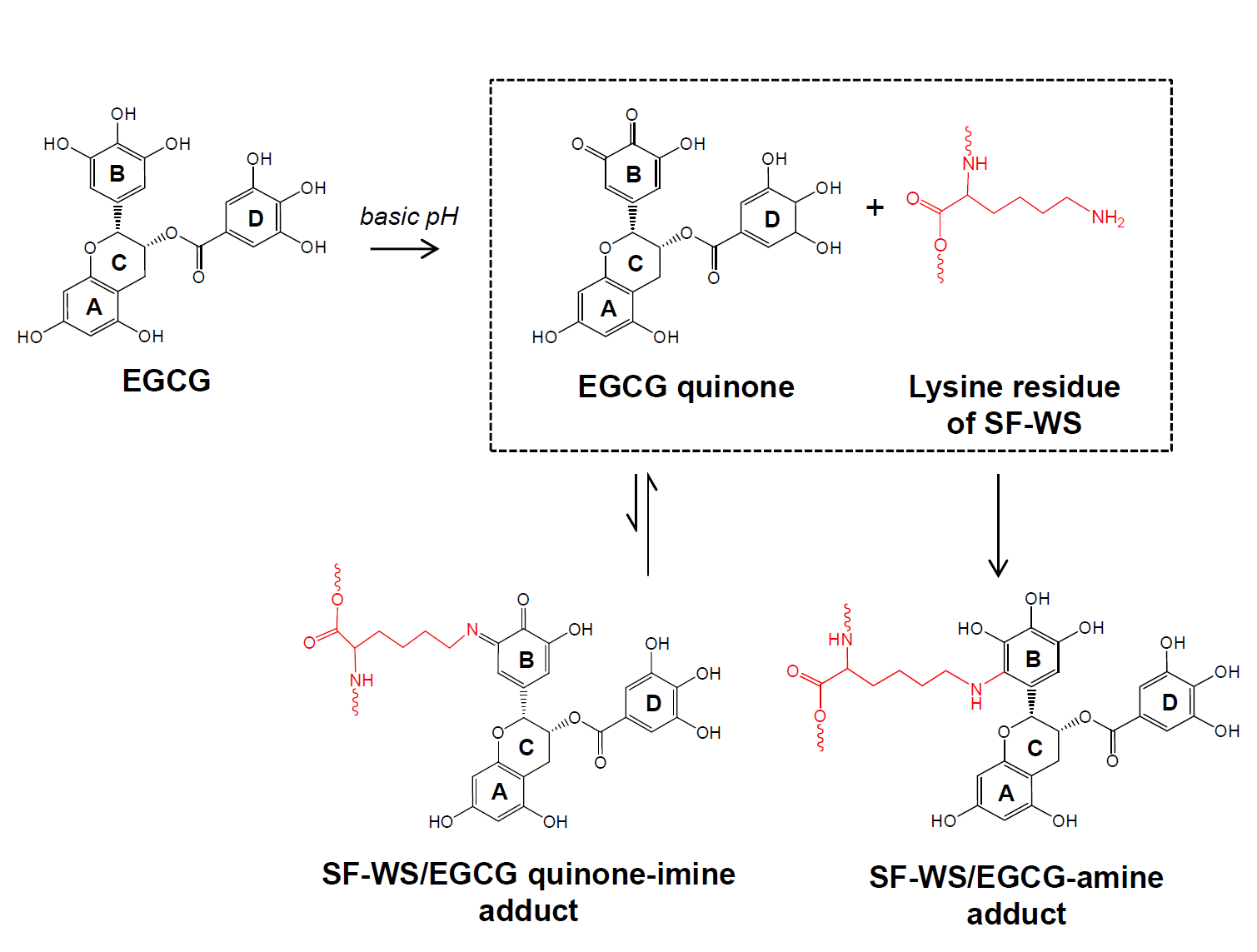


**Fig. S1.** The reaction mechanism for the SF-EGCG conjugates formation through autoxidation of EGCG at mild basic pH 7.4 and subsequent conjugation of EGCG quinone (ring B) with a lysine residue of SF-WS. SF-WS/EGCG-amine adduct (Michael addition) is considered the dominant final product as the minor formation of SF-WS/EGCG quinone imine adduct (Schiff base addition) is unfavorable due to rapid hydrolysis of the imine bond in aqueous solution.


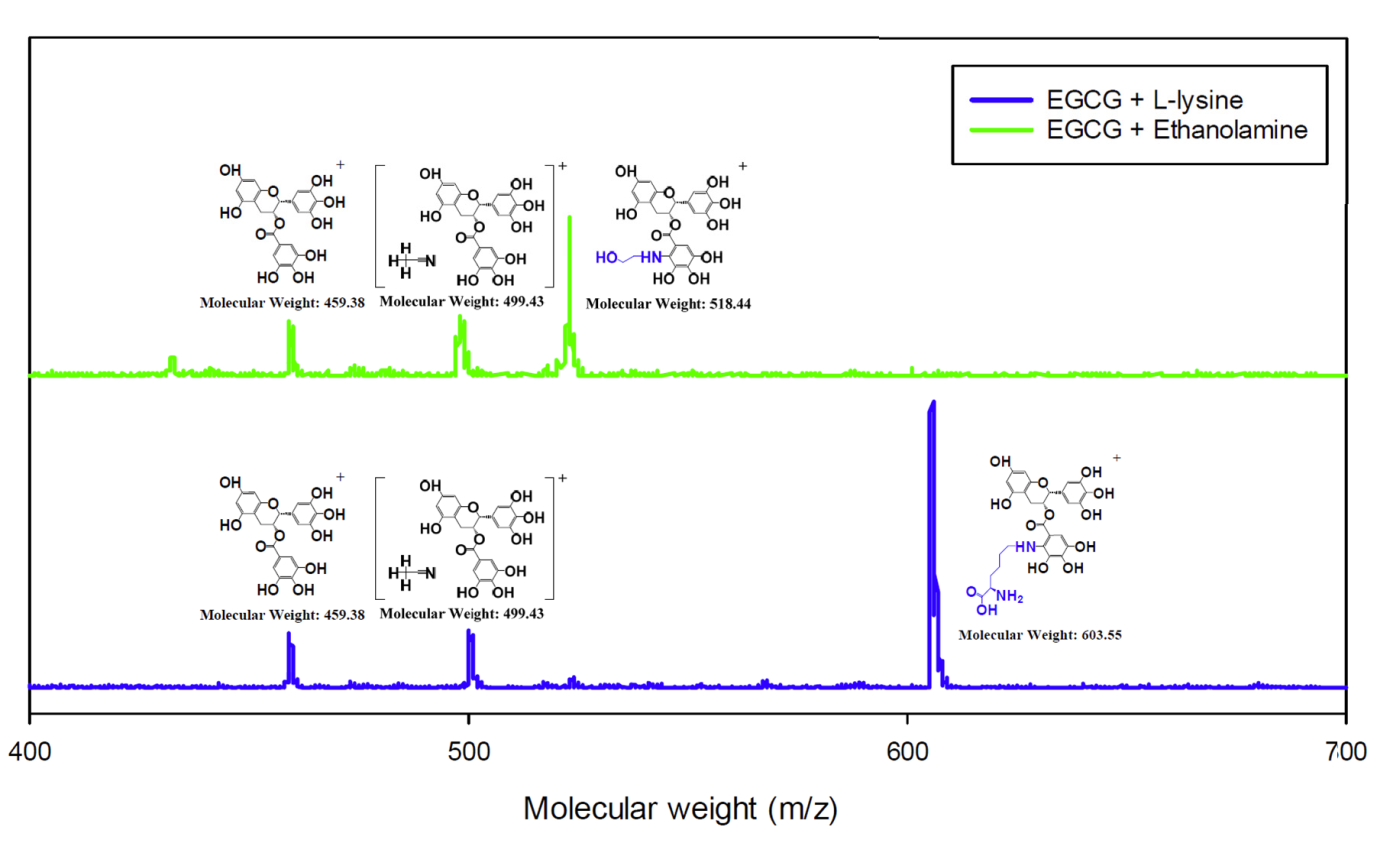


**Fig. S2.** Mass spectrum of EGCG-L-lysine reaction product and EGCG-ethanolamine reaction product.


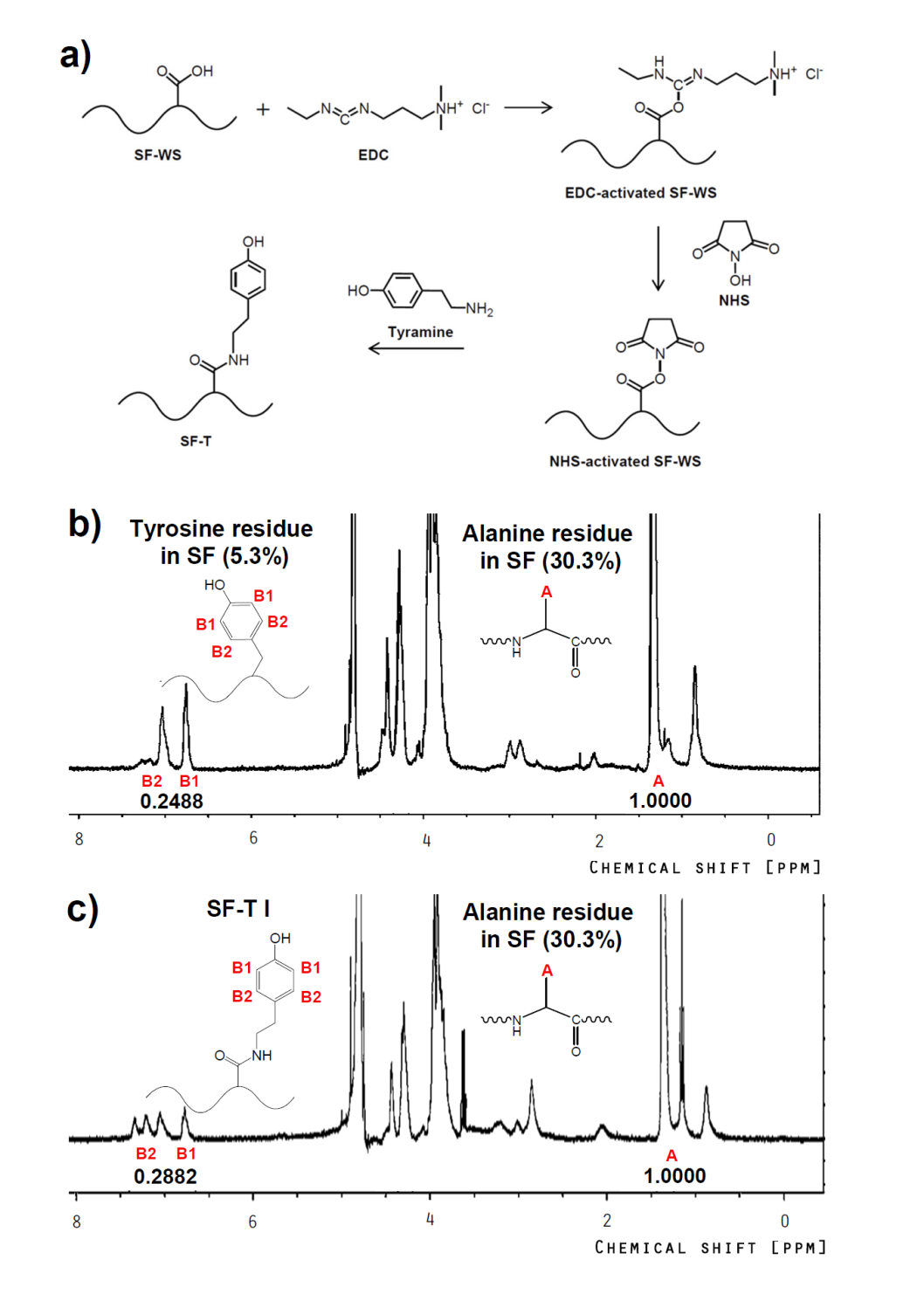


**Fig. S3.** (a) Synthetic scheme for SF-tyramine (SF-T) conjugate through EDC/NHS activation of carboxyl residue of SF-WS and subsequent amidation reaction with a primary amine group of tyramine. ^1^H NMR spectra of (b) SF-WS and (c) SF-T (DS = 1.37). The integrated intensity of the characteristic peaks of tyrosine and tyramine moieties (6.6~7.4 ppm, B1, B2) was compared with that of the peak of alanine residues (1.3~1.5 ppm, A) to determine DS of tyramine moieties.


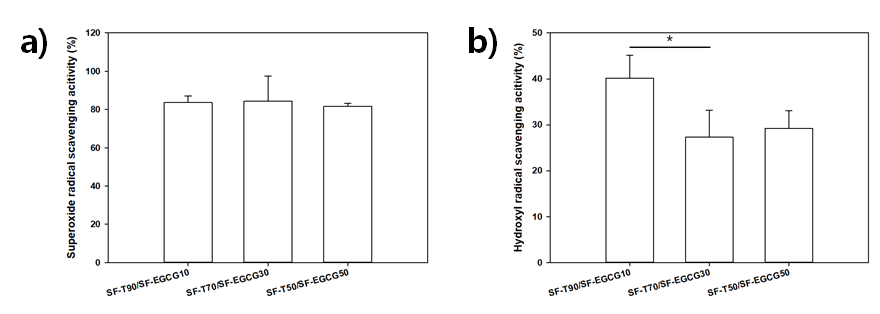


**Fig. S4. (**a) Superoxide anion radical (O_2_•¯) and (b) hydroxyl radical (•OH) scavenging activity (n = 3) of SF-T/SF-EGCG composite hydrogels.


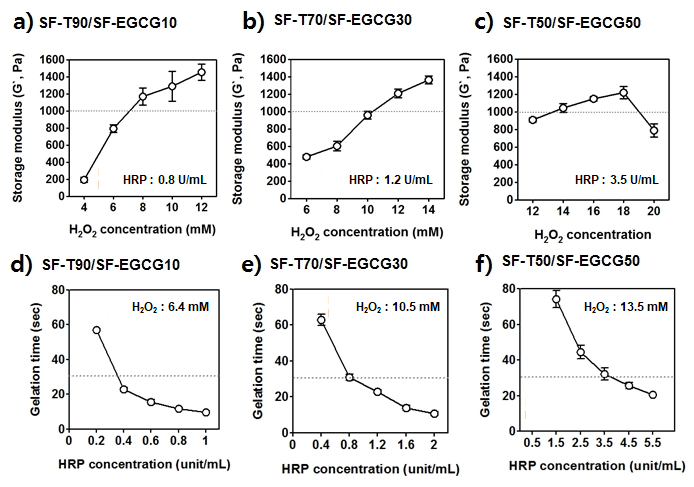


**Fig. S5.** Storage modulus of (a) SF-T90/SF-EGCG10, (b) SF-T70/SF-EGCG30 and (c) SF-T50/SF-EGCG50 composite hydrogels as a function of H_2_O_2_ concentration. The dotted line indicates the desired level of storage modulus (1,000 Pa). Gelation time of (d) SF-T90/SF-EGCG10, (e) SF-T70/SF-EGCG30 and (f) SF-T50/SF-EGCG50 composite hydrogels as a function of HRP concentration. The dotted line indicates the desired threshold for gelation time (30 sec), (Mean ± SD, *n* = 5).


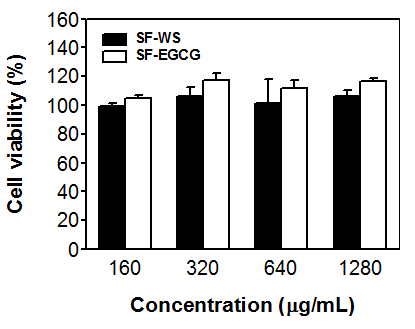


**Fig. S6.** Viability of NIH3T3 fibroblasts as a function of SF-WS and SF-EGCG concentrations for 24 hours (Mean ± SD, *n* = 4).


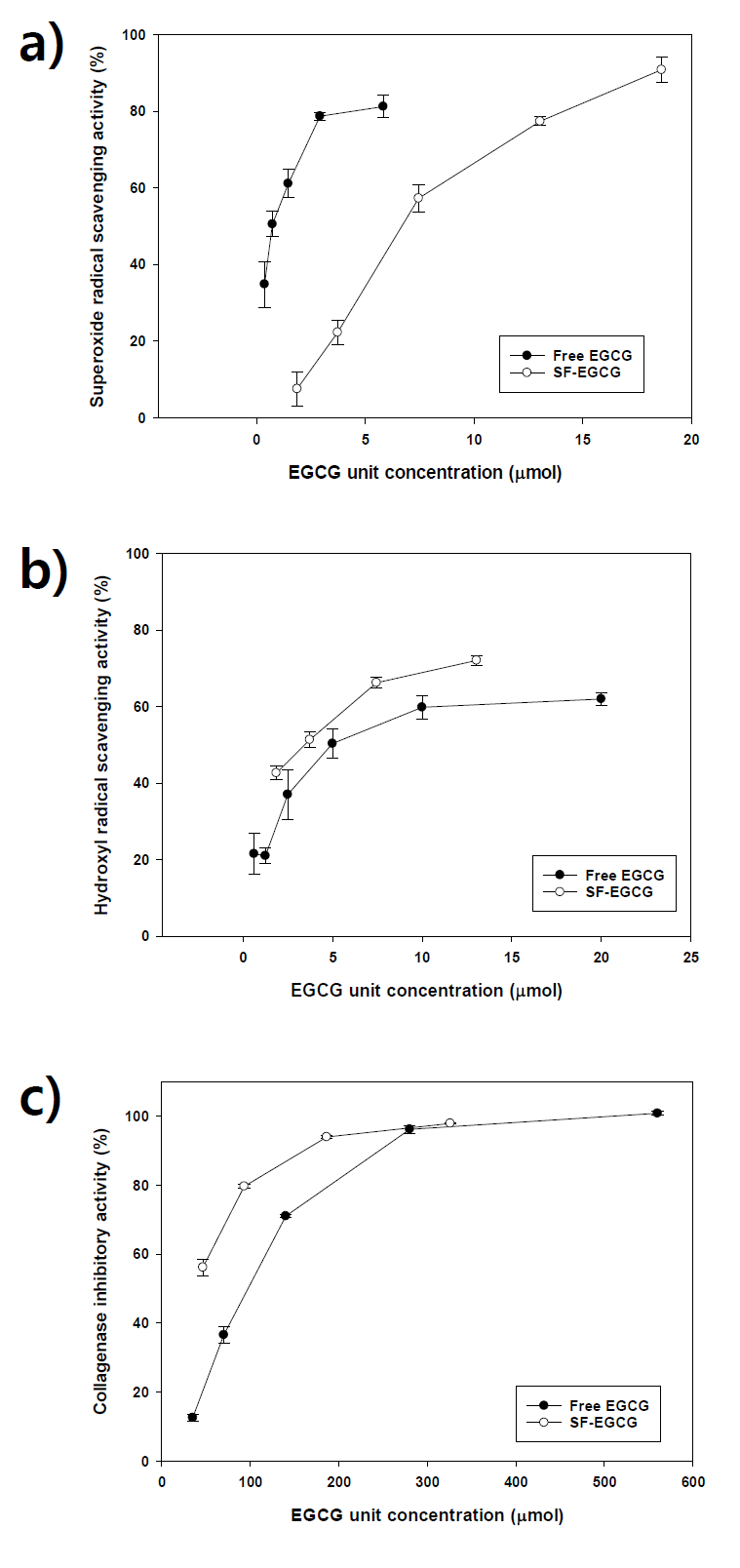


**Fig. S7.** (a) Superoxide anion radical (O_2_•¯) and (b) hydroxyl radical (•OH) scavenging activity (n = 3) and (c) collagenase inhibitory activity (n = 3) of free EGCG and SF-EGCG as a function of concentration..
